# Transient Urinothorax Following Nephrostomy Tube Placement and Percutaneous Nephrolithotomy: A Case Report

**DOI:** 10.7759/cureus.64607

**Published:** 2024-07-15

**Authors:** Zhaoqian Zhang, Xiao Li, Mei Yang

**Affiliations:** 1 Internal Medicine, St. Luke's Hospital, Chesterfield, USA

**Keywords:** left-sided pleural effusion, renal calculi (kidney stones), cystinuria, bilateral nephrostomy tube, percutaneous nephrolithotomy (pcnl)

## Abstract

Urinothorax is a rare complication of urological procedures. This report presents a case of a patient who developed urinothorax following nephrostomy tube placement and percutaneous nephrolithotomy (PCNL). The patient was managed conservatively with chest tube and Foley catheter placement, without the need for surgery. Computed tomography (CT) and chest tube output indicated that the urinothorax occurred immediately after nephrostomy tube placement but resolved within a couple of days without further intervention. Unlike some other cases that required surgical intervention due to persistent urine leakage, this case underscores the importance of prompt identification and tailored management of this rare condition based on clinical judgment.

## Introduction

Urinothorax is a rare clinical condition characterized by the presence of urine in the pleural space. This condition usually arises from urinary tract obstructions, trauma, or surgical procedures that permit urine to escape into the retroperitoneal or peritoneal cavity and subsequently reach the pleural space, either directly through diaphragmatic defects or indirectly via lymphatic pathways [1-3]. Accurate diagnosis and timely treatment are vital for patient outcomes, as the clinical presentation can closely resemble other causes of pleural effusions, such as infections, malignancies, or heart failure. Previously reported cases of urinothorax have often been treated with invasive surgical interventions, such as nephrostomy or surgical repair of the urinary tract. In contrast to this, we present a case of transient urinothorax where the patient was managed conservatively with chest tube placement and careful monitoring of pleural fluid drainage [4].

## Case presentation

A 34-year-old female with a history of recurrent nephrolithiasis presented to the emergency room (ER) with shortness of breath (SOB) after undergoing nephrostomy tube placement and PCNL.

**Figure 1:**
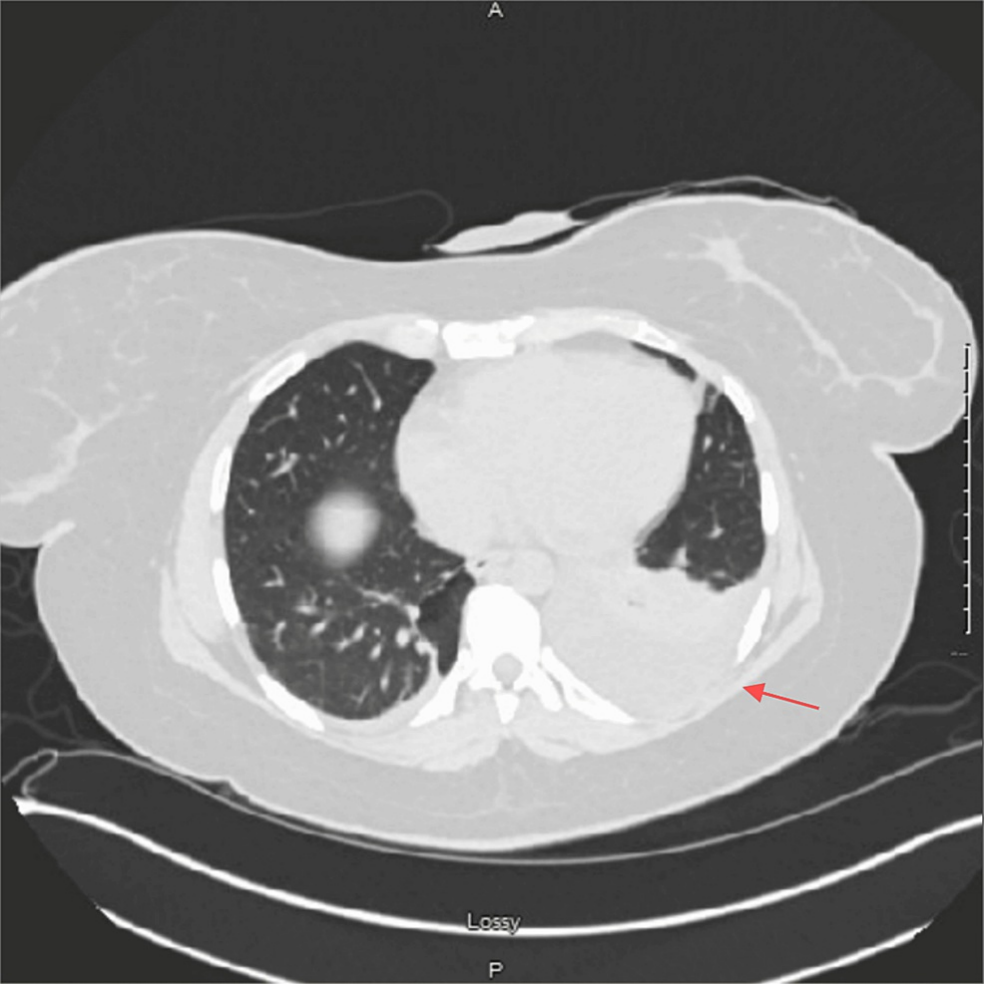
CT abdomen/pelvis without contrast The right arrow indicates left-sided pleural effusion.

The patient had a long-standing history of cystinuria and cystine kidney stones, which were well-controlled until her pregnancy when she had to discontinue tiopronin. Her bilateral kidney stones recurred and worsened, causing severe back pain. An ultrasound revealed an echogenic staghorn calculus in the left renal pelvis. She underwent successful nephrostomy tube placement and PCNL, followed by the removal of the nephrostomy tube. A post-procedural CT scan of the abdomen and pelvis revealed a left pleural effusion (Figure 1). On the following day, she developed worsening SOB and was admitted to the ER.

On admission, her labs revealed leukocytosis (Table 1).

**Table 1:** Lab results on admission The admission lab results are unremarkable.

Test	Result	Reference range
WBC (K/uL)	12.2	4-11
Neutrophil%	80%	40-60
Neutrophil (K/uL)	9.8	1.5-8
Hemoglobin (g/dL)	12	12-18
Platelet (K/uL)	331	130-400
Sodium (mmol/L)	137	135-145
Potassium (mmol/L)	4	3.5-5
Chloride (mmol/L)	108	95-105
Creatinine (mg/dL)	0.72	0.6-1.2
NT-proBNP (pg/mL)	357	<125

**Figure 2:**
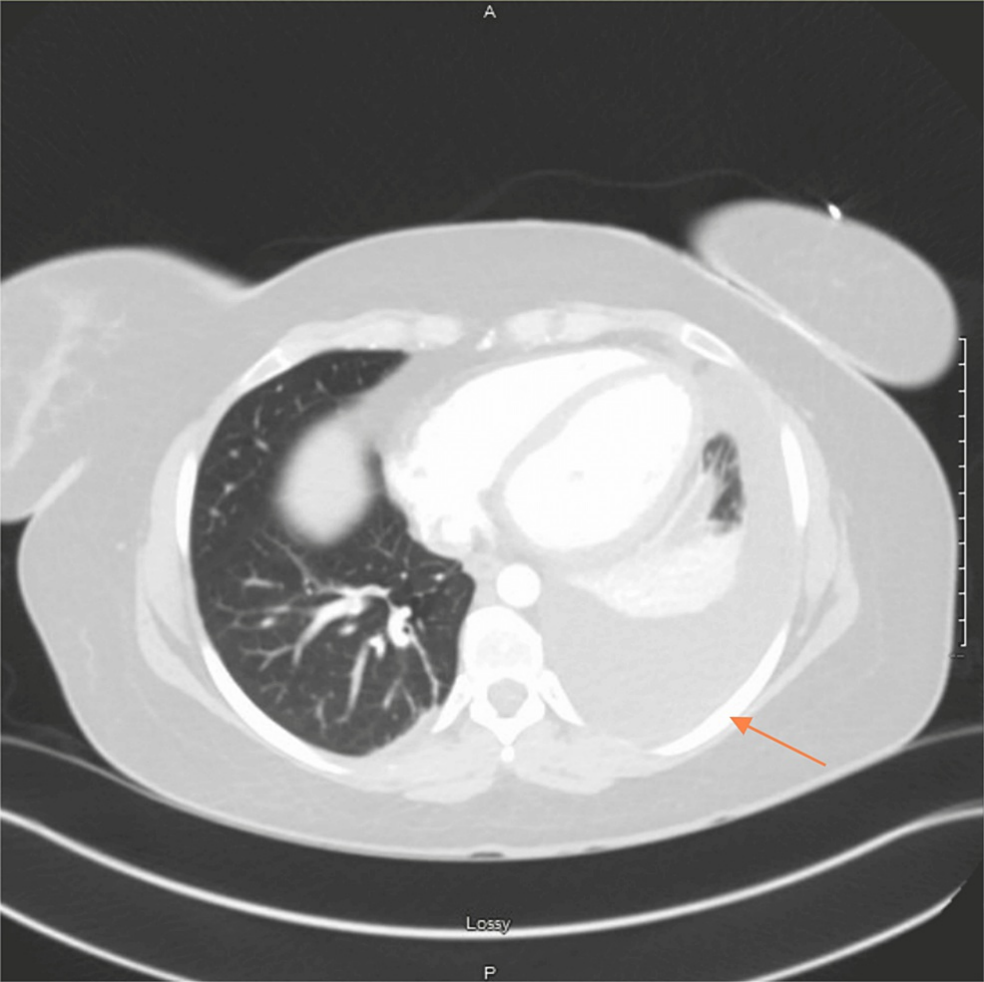
CT angiogram pulmonary embolism (PE) per protocol The orange arrow indicates worsened left-sided pleural effusion.

**Figure 3:**
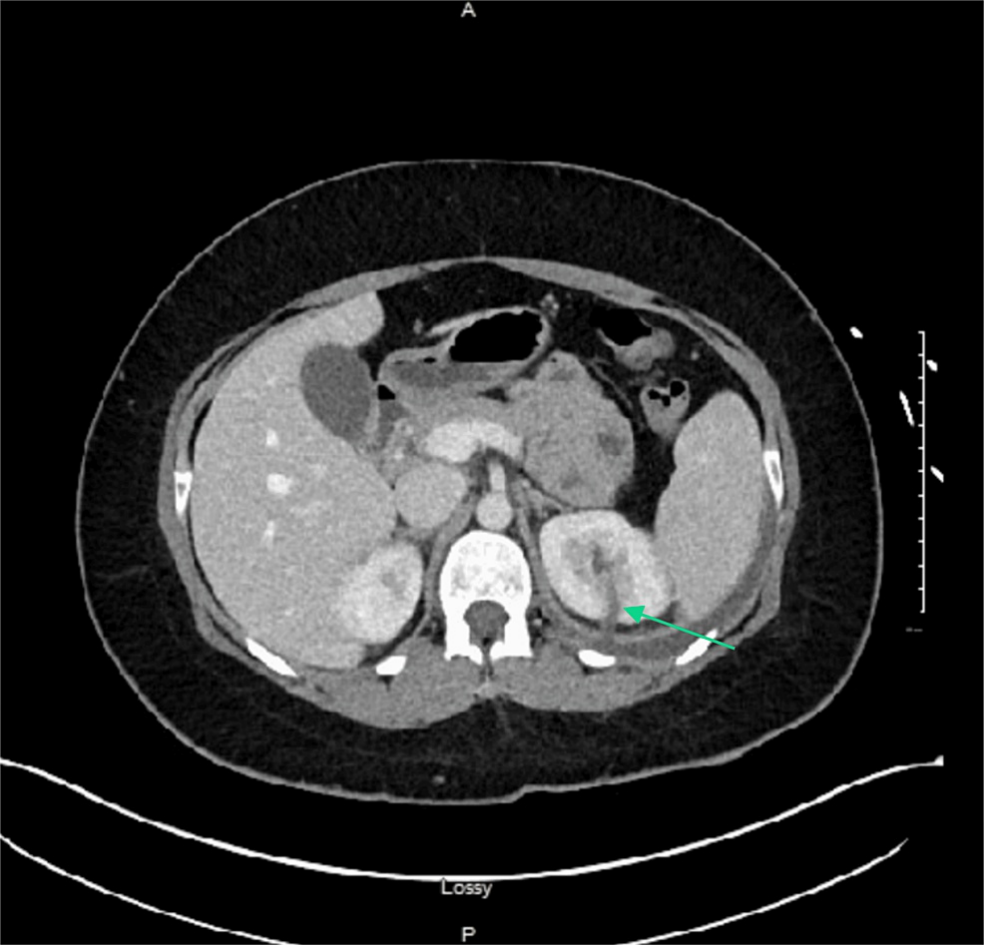
CT abdomen/pelvis with contrast. The green arrow indicates a posterior defect in the left kidney

A CT angiogram PE protocol and a CT abdomen/pelvis with contrast revealed no acute pulmonary embolus but showed a moderate to large left pleural effusion with compressive atelectasis that was worse than the previous CT. In addition, a posterior defect was identified in the upper pole of the left kidney, corresponding to the percutaneous access site for PCNL. There were no signs of active urine leak on the CT (Figure 2 and Figure 3).

Following consultation with urology and pulmonology, a chest tube and Foley catheter were placed. Pleural fluid analysis revealed elevated red blood cells, likely resulting from trauma during the PCNL procedure, along with a creatinine level of 3.57 and a pleural/serum creatinine ratio of 5.1, which is diagnostic for urinothorax (Table 2).

**Table 2:** Pleural fluid analysis Pleural fluid analysis indicated elevated red blood cells, creatinine (3.57), and a pleural/serum creatinine ratio of 5.1

Pleural fluid analysis	Result	Reference range
RBC	75000	<100
Glucose (mg/dL)	49	Similar to plasma
LD (U/L)	124	N/A
PH	7.41	7.60–7.66
Protein (g/dL)	<2	<2
Triglyceride (mg/dL)	<10	<10
Creatinine (mg/dL)	3.57	N/A
Pleural/serum creatinine ratio	5.1	<1

Due to the absence of active urine leakage into the pleural cavity, the patient was managed conservatively with chest tube placement and Foley catheter placement. The chest tube output was 1.6 L on the first day, 0.7 L on the second day, and 0.1 L on the third day. As the patient’s symptoms improved significantly, the chest tube was removed on the fourth day without complications. Cytological analysis of the pleural fluid showed reactive mesothelial cells with acute and chronic inflammation, no malignancy or infection. The patient was discharged without issues. During a three-month follow-up, the patient's kidney stones were found to be predominantly cystine, and she experienced a bilateral recurrence of kidney stones.

## Discussion

Urinothorax, the presence of urine in the pleural space, is an unusual condition [1-5]. The common causes include obstructive uropathy with hydronephrosis and diaphragmatic disruption due to blunt abdominal trauma. Other causes include percutaneous endoscopic renal procedures, retroperitoneal inflammatory processes, polycystic kidney disease, ureteral valves, extracorporeal lithotripsy, and intra-abdominal compression from a gravid uterus or lymphomatous masses [6-8]. The proposed mechanisms for urinothorax include direct extraperitoneal urine collection into the pleural space or lymphatic drainage of urine [8-10]. In this case, urinothorax likely developed from urine extravasation during and after the procedure, evidenced by the pinkish appearance of the urine and pleural fluid, and the high RBC levels in pleural analysis.

Diagnosis of urinothorax is confirmed via thoracentesis, revealing fluid with a urine-like odor and transudative nature with a pH lower than 7.30. A pleural fluid-to-serum creatinine ratio greater than 1 is diagnostic of urinothorax [11-14]. Other diagnostic tests may include renal scans, pyelograms, and contrast-enhanced CT scans to locate urinary system defects. Renal ultrasound and CT abdomen can show hydroureteronephrosis, and contrast-enhanced CT can reveal a reno-pleural fistula. Technetium-99m renal scintigraphy can demonstrate the translocation of 99Tc-labeled albumin from the genitourinary tract into the pleural space [11-18].

In this case, the pleural/serum creatinine ratio of 5.1 confirmed the diagnosis of urinothorax. The contrast-enhanced CT abdomen/pelvis showed no reno-pleural fistula, suggesting that the urine leak occurred shortly after nephrostomy tube removal. The chest tube outputs (1.6 L on the first day, 0.7 L on the second day, and 0.1 L on the third day) indicated a cessation of urine leakage within one to two days. Current management of urinothorax as cited in the literature often includes invasive surgical interventions, such as nephrostomy or surgical repair, to address the underlying cause and prevent recurrence. By contrast, our case highlights a successful conservative approach, utilizing chest tube placement and careful monitoring of pleural fluid drainage. The successful resolution of the patient's symptoms and subsequent discharge highlight the effectiveness of conservative management for transient urine leakage, indicating that non-invasive methods may suffice in similar clinical scenarios [18-20].

## Conclusions

This case report highlights the significance of recognizing urinothorax as a rare but potential complication following nephrostomy tube placement and percutaneous nephrolithotomy. Timely identification and appropriate management are crucial, as conservative treatment with chest tube and Foley catheter placement may suffice in cases of transient urine leakage. This case emphasizes the need for individualized clinical judgment to guide patient management, avoiding unnecessary surgical interventions. Increased awareness and understanding of urinothorax can lead to better diagnostic accuracy and patient outcomes in similar clinical scenarios.
